# Supplementary material for: Self-supervised contrastive learning improves machine learning discrimination of full thickness macular holes from epiretinal membranes in retinal OCT scans
Source: PLOS Digit Health. 2024 Aug 26;3(8):e0000411. doi: 10.1371/journal.pdig.0000411 (PMC11346922; doi:10.1371/journal.pdig.0000411)
Supplement: Attachment — Submitted filename: Response to Reviewers.docx [file pdig.0000411.s001.docx]

April 5, 2024

Dear Editor,

We would like to thank the reviewers for their efforts to review our manuscript. Below is a response to the individual suggestions of the two reviewers made in response to our submission. We hope that our replies to these suggestions and the manuscript revisions address each of these points in a clear manner.

Thank you.

**General Comments**

Have the authors made all data underlying the findings in their manuscript fully available (please refer to the Data Availability Statement at the start of the manuscript PDF file)? PLOS Data policy requires authors to make all data underlying the findings described in their manuscript fully available without restriction, with rare exception. The data should be provided as part of the manuscript or its supporting information, or deposited to a public repository. For example, in addition to summary statistics, the data points behind means, medians and variance measures should be available. If there are restrictions on publicly sharing data—e.g. participant privacy or use of data from a third party—those must be specified.

Answer: No | No

Reply: We apologize for this oversight during the initial submission process. The full dataset used for training and testing has now been uploaded to Harvard Dataverse, along with relevant demographic data (Table 1) to recapitulate data stratification in compliance with the Data Availability Statement.

**Reviewer 1 Comments**
**                Introduction:*
    ** Incorporate references in the introduction section to support the statements made. Several paragraphs lack proper citations.*

Reply: We agree with the reviewer and have added additional sources throughout the Introduction section to provide greater support. Both the Introduction and Reference sections have been updated to properly cite these new references. The complete Reference section has been updated and properly cites all sources throughout the manuscript.

    ** Clarify the rationale for specifically comparing ERM to full-FTMH. While ERM can lead to macular edema and retinal anatomy distortion, it is not clinically similar to FTMH. Discuss why this specific comparison was chosen.*

Reply: We agree that the rationale for FTMH and ERM comparison needs to be clarified in the manuscript. ERM and FTMH rationales have been added to the manuscript. This can be found in paragraphs 2, 3, and 4 of the introduction.

    ** Explore the clinical generalizability of the findings. Explain whether it makes clinical sense for an automated system to distinguish between ERM and FTMH, and why this distinction is relevant in practice.*

Reply: The relevant distinction in practice is explained in more detail throughout paragraph 3 of the introduction in addition to the clarification of FTMH vs ERM classification rationale. The benefit to automated classification is discussed both in the Introduction and in the last paragraph of the discussion including “streamlines clinical workflow, reduces clinician workload, and expedites referrals so that patients get access to specialist care sooner, improving overall outcomes.”

**                Patient Data:*
    ** Clearly describe the patient data selection process and its source. Emphasize any potential selection bias in the FTMH and ERM datasets, particularly since the data is derived from patients who underwent ocular surgery.*

Reply: We agree with the reviewer and have expanded the Materials and Methods section with a “Patient data” subsection to describe our patient data source and selection process. We address the possibility of selection bias by instituting a ground truthing process which involved three trained readers with exclusion criteria as follows: “Patients with non-idiopathic macular holes (traumatic, pseudohole), with history of ocular trauma, amblyopia, recent ocular surgery (within three months), severe ocular myopia, ~~any~~ diabetic macular retinopathy, and retinal pathology associated with systemic conditions (uncontrolled hypertension)”. Potential surgical bias is discussed in paragraph 5 of the Discussion; “our data is limited to patients who have undergone ILMx which may introduce selection bias in our dataset as this excludes patients who may have elected against surgical intervention, or patients lost to follow up”.

    ** Specify whether the research group excluded other macular pathologies besides diabetic maculopathy, and provide details on any other findings that were considered and present.*

Reply: We agree and have expanded on the macular pathologies under “Patient data” in the “Materials and methods” section with the following “Patients with non-idiopathic macular holes (traumatic, pseudohole), history of ocular trauma, amblyopia, recent ocular surgery (within three months), severe ocular myopia, diabetic macular retinopathy, and retinal pathology associated with systemic conditions (uncontrolled hypertension) were excluded”.

    ** I suggest adding race and ethnicity description.*

Reply: We agree that adding a race and ethnicity description is an important variable in analysis. Unfortunately, the original data collection did not account for these descriptions. The authors recognize that a balanced race and ethnicity distribution would be beneficial and discuss this limitation in paragraph 5 of the Discussion due to “a predominantly Caucasian patient population”.

    ** Were applied any quality control screening for OCT exams?*

Reply: We agree that quality control is an important aspect of data analysis, and have clarified our quality control methods in the “Patient data” subsection under Materials and Methods to add a quality control screening explanation including the assessment “by three trained readers to confirm diagnoses (FTMH [Gass stage 3 or 4] and ERM) and image quality”.

**                FTMH:*
    ** Specify whether the FTMH considered in this study is limited to idiopathic macular holes.  Even though some studies showed no effect on delay, the cited study (ttps://doi.org/10.1016/j.ophtha.2022.08.028) identified worse outcomes in postponed surgeries for idiopathic macular holes.*

Reply: We appreciate and agree with the review of idiopathic macular hole surgical delay and have included the patient data contained only “Patients with non-idiopathic macular holes” and discussed this further in the Methods and Materials section.

**    Macular holes have different classifications, such as the Gass, where stages 2-4 are full thickness but present differences. Add the Gass or IVTS classification for the included FTMH slabs.*

Reply: Agreed. The Gass stage classification of macular holes has been added to the manuscript. Gass stage classification is highlighted in the second paragraph of the introduction; “In Gass’ original classification [1, 38], the natural progression of macular holes proceeds through stages associated with worsening visual acuity: foveolar detachment in stage 1, formation of a foveal hole with associated subretinal fluid and cystoid macular edema in stage 2, formation of an FTMH in stage 3, followed by an FTMH with a complete posterior vitreous detachment from the vitreofoveal interface in stage 4. Due to involvement of the fovea, this results in impairment of central visual acuity [1]”. Our data collection consists only of patients with stages 3 and 4 macular holes and is stated within the first paragraph of the “Patient Data” subsection of “Materials and Methods”. As discussed in paragraph 2 of the “Patient Data” subsection this classification was also reviewed by three trained readers for confirmation.

**                Model Development:
    * On page 5, when discussing "Developing a functional deep learning model," clarify the definition of a "functional model”.*

Reply: We have amended our wording in reference to this to instead describe it as an “accurate deep learning model”, to enhance clarity.

**                Image-Level Description:
    * Include a comprehensive description of image-level findings, specifying the number of abnormal slabs detected in the images. This will provide a clearer understanding of the scope of abnormalities captured by the model.*

Reply: We believe the reviewer would like to know more detailed performance information at a patient level. We agree this is useful information, and we have modified the results section to include sensitivity, specificity, and precision across replicate experiments. We have also included heatmaps showing which portions of an input contribute to its output classification to explain image-level findings.

    ** I strongly suggest sharing the study data and codebase to enable validation and generalizability studies. In the data availability statement the authors state that “All relevant data are within the manuscript and its Supporting Information files”, but there is no supplemental file available, no available image data and metadata, and no code repository.*

Reply: We agree with the reviewer and apologize for the omission during the initial submission process. The Materials and Methods section has been updated with a GitHub repository including full model code (including for ImageNet-pretrained comparison) as well as code vignettes showing data preparation and processing. All data used in this study has been made available via a Harvard Dataverse repository, including relevant demographic metadata.

**Reviewer 2 Comments**

*Major Comments:*

*1. The absence of a data/code sharing statement significantly hampers the study's reproducibility. A clear statement regarding the availability of data and code is essential.*

Reply: We agree with the reviewer and apologize for the omission during the initial submission process. The Materials & Methods section has been updated with a GitHub repository including full model code (including for ImageNet-pretrained comparison) as well as vignettes showing data preparation and processing. All data used in this study has been made available via a Harvard Dataverse repository, including relevant demographic metadata.

*2. Adding a table with F1 scores per class and sample counts would provide a clearer understanding of the model's performance and enhance result transparency.*

Reply: We agree that clarity of statistics is important and have added a table (Table 2) with a full statistical summary of performance, including F_1_ scores and sample counts for FTMH and control classes.

*3. There is a notable lack of detailed methodology for the training and preprocessing of the baseline ImageNet pre-trained model, which is crucial for a fair comparison.*

Reply: We agree, and the Materials and Methods section has been updated with information regarding the ImageNet pre-trained model. The code used to run this model has also been made available in the GitHub repository.

*4. Incorporating a figure of the proposed method would aid in understanding the complex methodology and enhance clarity.*

Reply: We agree with the reviewer and understand the importance of including a visual aid to explain the training model and a visual overview of the proposed method has been added to the Self-supervised pre-training subsection under Materials and Methods.

*Minor Comments:*

*1. The discussion should not only address the strengths of the paper but also delve deeply into the implications and limitations. The size of the dataset and possible biases due to sex imbalance should be discussed.*

Reply: We agree with the importance of addressing study limitations as well as the importance of equal sex distributions within a data set. The sex imbalance has been addressed in paragraph 5 of the Discussion section: “To develop higher confidence in the model for deployment, other devices (*e.g.,* Zeiss Cirrus, and Topcon), a patient population with a broader demographic profile, a balanced sex distribution and disease-agnostic OCT data would be required”. Additional limitations have also been discussed in more detail in the same paragraph. Paragraphs 1-4 of the Discussion address dataset size limitations.

*2. A more critical comparative analysis between the proposed model and the baseline in the discussion section would strengthen the manuscript.*

Reply: We agree with the reviewer that a strong comparative analysis is important in elucidating the benefits of the new methodology. We have provided a comparison of specific performance metrics in comparison with the baseline model (Table 2). We have also highlighted the heatmaps as a visual aid in presenting explainable algorithm outputs.

*3. Emphasis on methodological rigor and transparency across all experimental procedures is paramount for the scientific validity of the research.*

Reply: We agree with the importance of methodological rigor and transparency for experimental procedures. The authors have expanded the Materials and Methods section to create a more transparent overview of the data selection and training process. The data release to Harvard Dataverse increases the study transparency for data collection and the sharing of code permits fellow researchers to replicate our results.

*4. The initial portion of the Discussion section, which provides background information, should be relocated to the Introduction to better set the context for the study.*

Reply: We agree that moving this paragraph to the Introduction provides better context for the study and have done so.

*5. The decision to utilize an ImageNet pre-trained model as baseline, over domain-specific models such as Retfound (https://www.nature.com/articles/s41586-023-06555-x), necessitates a thorough justification.*

Reply: We agree with the reviewer that the RetFound model is a significant advance for the domain of ophthalmological deep learning. However, our manuscript was in the final stages of submission when this foundational model was published. In the 2^nd^ paragraph of the Discussion, we explain the options for dealing with low dataset size. The first is transfer-learning (ImageNet pre-training); the second, self-supervised contrastive pre-training (the presented method); and the third is to fine-tune a foundation model. A comparison of the third option to the other two is left to future work.

*6. The manuscript's structural organization needs refinement, including the incorporation of a conclusions section.*

Reply: As per PLOS Digital Health’s manuscript preparation guidelines, our Conclusions section was included in our Discussion section. <https://journals.plos.org/digitalhealth/s/submission-guidelines>
